# Reirradiation of recurrent breast cancer with and without concurrent chemotherapy

**DOI:** 10.1186/1748-717X-3-28

**Published:** 2008-09-18

**Authors:** Florian Würschmidt, Jörg Dahle, Cordula Petersen, Claudia Wenzel, Matthias Kretschmer, Christoph Bastian

**Affiliations:** 1 GMP Radiologie & Radioonkologie im Struensee-Haus, Mörkenstr. 47, D-22767 Hamburg, Germany; 2 HOPA, Hamburg, Mörkenstr. 47, D-22767 Hamburg, Germany

## Abstract

**Background:**

Treatment options for loco-regional recurrent breast cancer after previous irradiation are limited. The efficacy of chemotherapy might be hampered because of impaired tissue perfusion in preirradiated tissue. Thus, mastectomy or local excision and reconstructive surgery are the preferred treatments. However, in recent years evidence accumulates that a second breast conserving approach with reirradiation as part of the treatment might be feasible and safe and, furthermore, reirradiation might be an option for palliation. Here we report on the experience of a single community centre in reirradiation of recurrent breast cancer.

**Methods:**

The report is based on 29 patients treated with reirradiation. All data were prospectively collected. The median age was 63 years (range 35 to 82 yrs). The interval between initial diagnosis and diagnosis before start of reirradiation was 11.6 months to 295.5 months. The mean total dose (initial dose and reirradiation dose) was 106.2 Gy (range 80.4 to 126 Gy) and the mean BED_3 Gy _168,5 Gy (range 130,6 to 201,6). The mean interval between initial radiotherapy and reirradiation was 92.9 months (range 8.7 to 290.1). Inoperable or incompletely resected patients were offered concurrent chemotherapy with either 5-FU or capecitabine. All patients received 3D-conformal radiotherapy with 1.6 to 2.5 Gy/fraction five times weekly. The treatment volume comprised all visible lesions or lesions detectable on CT/MRI/FDG-PET/CT or the tumour bed or recurrent tumour.

**Results:**

The local progression-free survival of all patients at one and two years was 81% and 63%. Patients who had no surgery of the recurrence (16/29) had local progression-free survival at one and two years of 72% and 25% with a median progression-free survival time of 17 months. Partial remission and good symptom relief was achieved in 56% (9/16) or complete response of symptoms and/or tumour in 44% (7/16). Patients who had no distant metastases and had at least an R1-resection had a local progression-free survival of 90% after 2 years. The disease-free survival after 2 years was 43% and the median disease-free survival time was 24 months. In four patients a second breast conserving operation was performed and the cosmetic results in all four patients are good to excellent. Acute side effects were mild to moderate with no grade 3 or 4 toxicity. Accordingly, no grade 3 or 4 late effects were observed so far. No grade 3 or 4 plexopathy was observed.

**Conclusion:**

In this heterogeneous group of patients reirradiation of locoregional recurrences of breast cancer showed low to moderate acute toxicity. In our experience, local control rates are high and palliation is good.

## Background

Increasing numbers of breast cancer patients are treated with breast conserving surgery followed by systemic therapy and radiotherapy. Failure rates after breast conserving therapy (BCT) increase with 1–2% per year as reported by large centres [1-4] and even after mastectomy local recurrence rates might be as high as 40% depending on risk factors [5]. The treatment options for loco- regional recurrences are limited. Chemotherapy might not be effective in pre-irradiated tissue because a decreased perfusion can be expected due to radiation-induced fibrosis [6]. Mastectomy or local excision and reconstructive surgery are, thus, the preferred therapies. However, in recent years evidence accumulate that a second BCT might be feasible with long term local control. If cure is no longer achievable, local regional recurrences might cause suffering due to severe pain, bleeding and ulcerations in up to 62% of patients [7]. Furthermore, a growing tumour mass can be a stressful experience to a patient.

Retreatment with a second full course of radiation to the whole breast is used with caution as increased toxicity of skin and subcutaneous tissue is feared. Nevertheless, in recent years several investigators reported on reirradiation either alone or combined with concurrent hyperthermia or chemotherapy. Irradiation was applied as external beam therapy, brachytherapy or intraoperative radiotherapy.

The following report presents the experience of a single community centre with retreatment of loco-regional breast cancer recurrences after previous irradiation to the breast, chest wall, and/or regional lymphatic nodes. 3D conformal radiotherapy with or without concurrent 5-FU/capecitabine chemotherapy was given for operable and non-operable recurrences.

## Methods

Since 2003 we offer patients reirradiation of recurrent breast tumours based on recommendations of an interdisciplinary tumour board panel of gynaecology, medical oncology, diagnostic radiology and radiation oncology experts. This report is based on 29 patients treated with reirradiation. All data were prospectively collected. Written informed consent about the reirradiation procedure was given by all patients.

In 16 patients retreatment was offered because of bleeding, exulcerating and/or painful breast and/or local lymph node recurrences. Usually the patients had simultaneous distant metastases. No surgery of these recurrences was attempted except in one case with tumour debulking surgery. Usually, these patients had had multiple re-operations, chemotherapeutic or endocrine therapies and radiation treatment for distant metastases. Thirteen patients had no signs of distant metastases and surgery of the recurrences was done in all cases. The mean follow-up was 13.7 months (range 1.8 to 55.9 months) for all patients. Normal tissue toxicity was recorded according to the CTCAE (formerly known as CTC) v3.0 scoring system (Version 3.0; ).

### Patient characteristics

The median age was 62 years (range 35 to 82 yrs). The median interval between initial diagnosis and diagnosis before start of reirradiation was 70 months (range 10.3 to 295.5. Further details of tumour stage and recurrence site are given in table 1.

**Table 1:** Recurrent breast cancer treated with reirradiation.

**Pat**.	**Age**	**TNM initial diagnosis**	**TNM reirradiation**	**Recurrence site**	**Interval (months)**
2	67	pT2pN0M0	rT2R1G2M0	Infraclav., subpectoral right	137,4
6	63	pT2pN1M0	rpT2(m)R1NxL1M0G3	left thoracic wall	25,2
7	46	pT1R1pN0G3M0	rpT2RxM0	Caudal part of TRAM flap	225
9	65	pT2pN1G3LOV0	rpT1rpN3G3M0 R1	Intramamm., IMC, v. subclavia	31,3
15	66	pT2pN1M0	rpT2R1G3 cN0 M0	Left thoracic wall	164,8
18	78	pT1bpN3G3R0M0	M1 (SKI) R1 G3	Lymphang. carc. left thoracic wall	10,3
19	43	pT1bpN1G2L1R0	rpN2 R1 G2 M0	Left axilla, lateral thoracic wall	49,0
23	58	pT1R1G2 pN0M0	rpT1c rpN R0 G2 M0	Mastectomy scar	137,4
24	50	pT3pN1G3	rcN3 M1 (LYM)	LN level I-III, upper mediasti.LN	50,7
25	62	pT1bpN0G2	rpT1b G2 R0 pN0 M0	Lower left quadrant	215,8
26	71	pT3pN0M0 R1	rpT2 G3 R0 M0	Medial part of left thoracic wall	295,5
27	82	pT1pN1 G1M0	rcT4 cN0 M0	Right lower inner quadrant	105,5
28	44	pT1 G2 pN0 M0	rpT1b R0 G2 cN0 M0	Right upper outer quadrant	179,9
1	67	pT1pN0Mo	rcT4NxM0	Thoracic wall, extensive	181,7
3	44	pT2pN1G3	rcT4M1	Thoracic wall, extensive	97,8
4	49	pT1cpN0G3	rpT2RxG3pNxM1	Thoracic wall + axilla	13,1
5	35	pT3G3pN1M0	rpT4G3N3M1	Left toracic wall, cutan. metast	21,1
8	54	pT1pN0M0G3	M1	Parasternal recurrence	79,0
10	63	pT4pN1M0	rcN3M1	Thoracic wall + axilla	56,0
11	80	pT2pN0G2R0	rpT4 R2 cN0M0	Lateral thoracic wall + axilla	70,7
12	78	pT4bpN3M0G3R0,	rcN3M1	Skin metast., LN supra., ax.	11,6
13	71	pT1b GII pN1 M0	rcT4 N3 M0	Ax., supra. LN, mammary gland	50,6
14	49	ypT1ypN1G3L1	rcN3M1	Brachial plexus, skin metast.	39,2
16	61	pT1pN1M0G2	rcT4 M1	Sternal and parasternal	190,3
17	49	pT1pN2M0	rcN3cM1	Left supra., cervical LN	243,2
20	46	pT3 pN1 M0 G3	rcT4 rcN3	Thoracic wall with ulceration	41,2
21	70	pT2pN0M0R0	rcT4Mx	Thoracic wall, extensive	55,6
22	44	cT4cNxM0	rcT4Mx	Thoracic wall, extensive	83,9
29	79	pT1mpN2aM0	rcT4 cN0 M0	Thoracic wall, extensive	36,8

Pat.: patient number; IMC: internal mammary chain; LN: lymph nodes.

Clinical characteristics of recurrent breast carcinoma patients treated with reirradiation. The initial and recurrence TNM staging and site of recurrence are shown. The interval between the initial diagnosis and diagnosis before start of retreatment is given in months.

### Retreatment and statistical methods

Resection was incomplete or undefined (R1 or Rx) in 7/29. One patient had inoperable axillary and upper mediastinal lymph node metastases and one patient received preoperative reirradiation followed by mastectomy (R0). One patient had tumour debulking and axillary dissection before radiotherapy. All other patients were inoperable due to tumour extent and/or simultaneous distant disease.

All patients underwent computed tomography (n = 27) or FDG18-PET/CT (n = 2) for 3D-conformal radiotherapy planning. The dose per fraction was 1.6 to 2.5. The mean *total dose *(initial dose and reirradiation dose) of all patients was 106.2 Gy (range 80.4 to 126 Gy) and the mean total BED_3 Gy _168,5 Gy (range 130,6 to 201,6).

The Biologically Effective Dose (BED) was calculated as follows:

BED = (n*d)*(1 + d1/(α/β))

where n*d denotes the total dose and *d *the dose per fraction. α/β was assumed to be 3 Gy for late-responding tissues. The BED is the quantity by which different fractionation regimens can be compared. [8]

The mean interval between initial radiotherapy and reirradiation was 92.9 months (range 8.7 to 290.1). The *treatment volume *comprised all visible lesions or lesions detectable on on CT/MRI/FDG-PET/CT or the tumour bed or recurrent tumour. Treatment details with indication of the overlapping treatment volumes are shown in table 2.

**Table 2:** Recurrent breast cancer treated with reirradiation.

**Pat**.	**Initial dose Gy**	**Reirrad. Dose (Gy)**	**Dose 1 +2 Gy**	**BED_3 Gy_**	**Interval (m)**	**Overlapping treatment volume**	**Chemo-therapy**	**Surgery for recurrence**
2	55	50,4	105,4	178,1	133,6	Subpectoral + infraclavicular	5-FU	Segmental resection (BCT)
6	60,4	56	116,4	182,5	20,4	Left thoracic wall	none	mastectomy, ax. diss.
7	60	51,2	101,2	188,5	220,5	Caudal thoracic wall	5-FU	Local excision
9	45	54,4	99,4	155,4	26,8	Cranial thoracic wall	none	mastectomy, LN
15	60	51,2	111,2	198,5	161,5	Mastectomy scar	none	Thoracic wall resection,
18	50	57,6	107,6	175,5	8,7	lateral thoracic wall, ax.	none	Local excision
19	60,4	61,2	121,6	194,6	42,8	upper outer quadrant, ax.	Taxotere	resection, ax. diss. (BCT)
23	60	50,4	110,4	180,6	135,1	Left thoracic wall	none	reconstructive surgery
24	60,4	55,8	116,2	185,9	42,5	Mastectomy scar	Capecitab.	None
25	60	50,4	110,4	180,6	212,6	Left lower quadrant	none	Local excision (BCT)
26	50	50,4	100,4	172,3	290,1	Left thoracic wall	none	Thoracic wall resection
27	50	46	96	160,0	114	Right lower inner quadrant	none	Mastectomy post- reirradiation
28	60	59,4	119,4	195,0	175,0	Right upper quadrant	none	wide excision (BCT)
1	50	50,4	100,4	164,0	184,7	Lateral thoracic wall	none	none
3	50,4	30	80,4	130,6	32,5	Thoracic wall	Capecitab.	None
4	60	45	105	172,0	23,5	Thoracic wall	Capecitab.	None
5	64	44,8	108,8	175,4	52,6	Whole thoracic wall	Capecitab.	None
8	60,4	50	110,4	180,0	85,9	parasternal	none	None
10	50,4	57,6	108	169,0	50,2	Thoracic wall	5-FU	None
11	66,6	59,4	126	201,6	69,5	Lat.thoracic wall	none	None
12	50,4	45	95,4	152,6	8,7	Thoracic wall, lateral	none	None
13	60,0	50,4	110,4	180,6	49,9	Upper quadrants	none	None
14	60,4	50,4	110,8	177,3	28,7	Upper outer quadrant	5-FU	none
16	55	49	104	186,6	185,2	parasternal	none	None
17	42,5	45	87,5	149,9	201,2	supraclav., apex ax.	none	None
20	50	48	98	163,3	32,7	Sternal + infraclav.	Capecitab.	None
21	59,4	44	103,4	168,4	53,1	Thoracic wall	none	None
22	59,4	46	105,4	177,7	20,0	Thoracic wall	none	None
29	50,4	50	100,4	164,0	31,1	Thoracic wall	none	None

Pat.: patient number ; BCT: breast conserving therapy for recurrence; LN: lymph nodes; ax. diss: axillary dissection; capecitab.: capecitabine.

Treatment details of recurrent breast carcinoma patients treated with reirradiation. Radiation doses (Gy) of initial radiotherapy and reirradiation, the cumulative dose (dose 1 + 2) and the cumulative BED_3 Gy _(Gy) are shown. For the initial treatment, whole breast plus boost dose is given, if retreatment volume included the initial boost volume. The interval between the last day of the initial radiotherapy and first day of reirradiation is given in months.

Inoperable or incompletely resected patients were offered concurrent chemotherapy with either 5-FU (225 mg/sqm continuous infusion, seven days per week, during whole course of reirradiation) or capecitabine (1250 or 1650 mg bid during whole course of reirradiation) except in one case where docetaxel was given concurrently. All patients received chemotherapy *not *because of distant metastases but with the aim of an additional local effect. The minimum and maximum duration of chemotherapy was 3 weeks (with 30 Gy of reirradiation) and 5 weeks (reirradiation dose of ≥ 50 Gy). No chemotherapy was given to R0-resected patients. Due to multiple courses of chemotherapy because of metastatic disease and/or age and co-morbidities or refusal of patients not all could be treated with simultaneous chemotherapy. Endocrine therapy was given according to hormonal receptor status, i.e. premenopausal women received tamoxifen and postmenopausal women either an aromatase inhibitor or tamoxifen. Further details of retreatment for each patient are depicted in table 2.

Patients were seen on follow-up visits 6 to 8 weeks after end of reirradiation and thereafter every 4 to 6 months. All other patients were contacted by telephone or their gynaecological and medical oncologist were interviewed.

Kaplan-Meier survival curves were generated with GraphPad Prism 5.0 (^©^1992–2007 Graph Pad Software Inc.).

## Results

**Figure 1:**
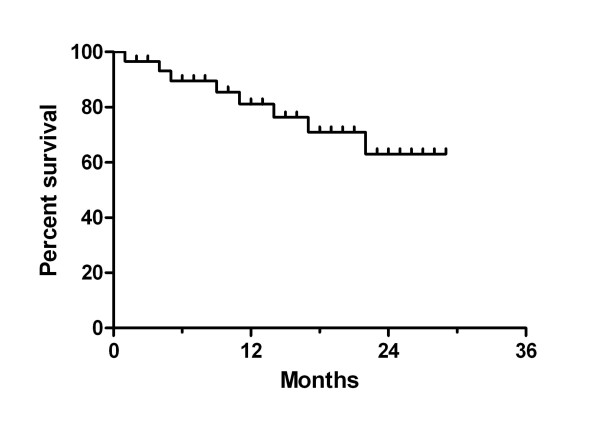
**Local progression-free survival of reirradiated breast cancer patients (months after start of retreatment; n = 29).** A heterogeneous group of patients were retreated with operable (R1 or R0 resection) and non-operable recurrent breast tumours with and without distant metastases. Reirradiation was given alone or combined with simultaneous 5-fluoruracil or capecitabine to loco-regional breast cancer recurrences after previous irradiation to the breast, chest wall, and/or regional lymphatic nodes.

**Figure 2:**
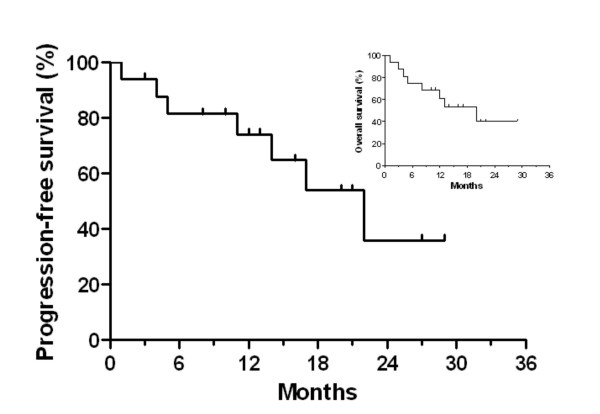
**Reirradiation of patients with non-operable recurrent breast carcinoma (n = 16).** Patients had either no surgery or only tumour debulking (n = 1). Local progression-free survival is shown (months after start or reirradiation). The inset depicts the overall survival percentage.

In figure 1 local progression-free survival is shown. The reported percentages indicate the local progression-free survival within the retreated volume (i.e. breast, chest wall, or supraclavicular/axillary lymph nodes). At one and two years the local progression-free survival was 81% and 63%. Those patients who had reirradiation because of non-operable recurrences (16/29) had local progression-free survival at one and two years of 72% and 25% with a median local progression-free survival time of 17 months (figure 2). Partial remission and good symptom relief was achieved in 56% (9/16) and complete response of symptoms and/or tumour in 44% (7/16). Nine of sixteen patients were free of local progressive disease during their lifetime. Overall survival times after one and two years were 61% and 40% and the median overall survival time was 20 months (inset of figure 2).

**Figure 3:**
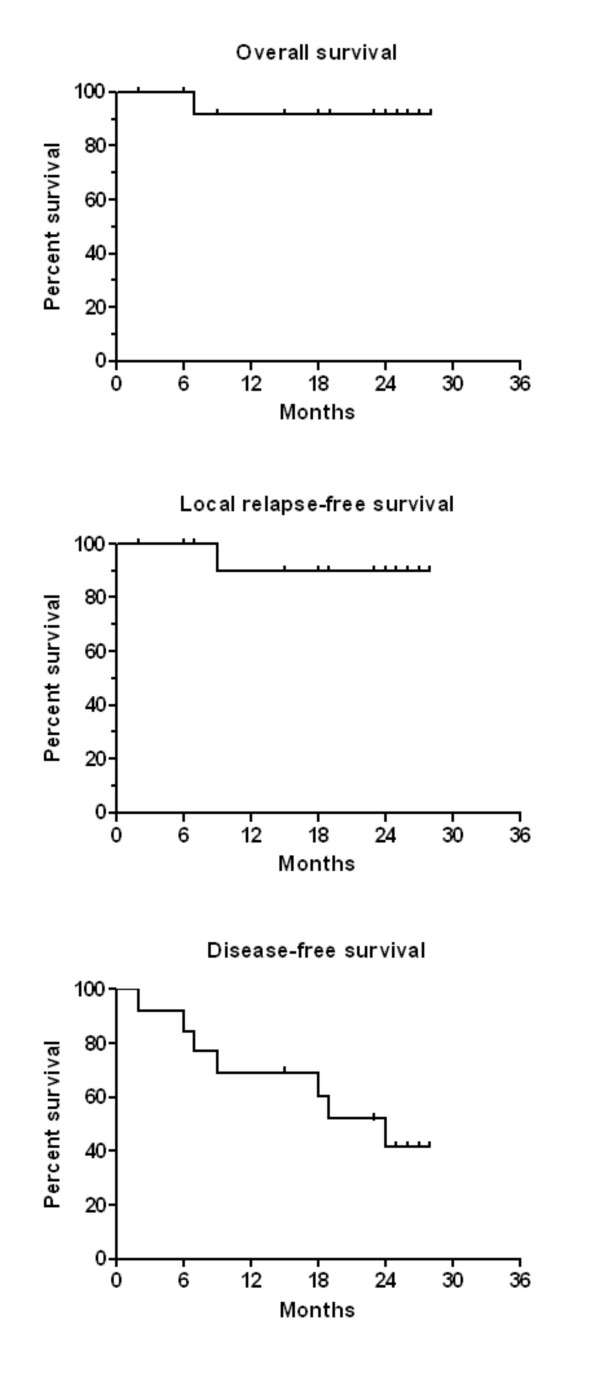
**Reirradiation of recurrent breast carcinoma. Survival proportions of a subgroup of patients (n = 13) are shown who were free of distant metastases at the time of diagnosis of the recurrence and who were operable (R0 or R1-resection).** Kaplan-Meier curves for overall survival (panel A), local relapse-free survival (panel B), and disease-free survival (panel C) are given.

The survival times of patients who had no distant metastases and who at least had a macroscopically complete resection (R1) of the recurrence are given in figure 3. The overall survival (figure 3A) was 92% and the local relapse free survival 90% (figure 3B) after 2 years. One patient (no. 8) had a local (infield) relapse in infra- and supraclavicular lymph nodes 19.7 months after reirradiation with 54,4 Gy (cumulative dose 99,4 Gy). She developed simultaneous lymph node metastases in the contralateral axilla. The relapse was documented on FDG-PET/CT and histological proven. As she was free of symptoms the patient decided to have no further treatment and is alive 11 months after the relapse (30,7 months after start of reirradiation). A second patient (no. 19) developed a simultaneous inflammatory recurrence of the whole breast and bone metastases 15.8 months after start of reirradiation. She is alive and receives chemotherapy.

The disease-free survival after 2 years was 42% and the median disease-free survival time was 24 months (figure 3C). One patient had intracerebral metastases which were treated with stereotactic radiotherapy. She died 28,5 months after reirradiation with no signs of a local recurrence within the reirradiated breast. One patient (no. 2) had multiple bone and cerebral metastases 8,5 months after reirradiation. She is alive with disease 26,5 months after reirradiation. One patient suffered from a contra lateral breast cancer 20.6 months after reirradiation and skin metastases. She was treated with trastuzumab and a taxane and is alive 31,8 months after reirradiation. One male patient with a recurrent breast cancer (no. 18) had skin metastases outside the retreatment volume at the medial parts of the thoracic wall and upper abdominal wall 6,9 months after reirradiation. He received taxanes and had a complete response. He is alive 15,2 months after reirradiation.

In three patients a second breast conserving operation was performed and the cosmetic results in all three patients are excellent (1) or good (2).

### Normal tissue toxicity

Acute side effects were mild to moderate (skin erythema grade 1 to 2) in *all *patients with no grade 3 or 4 toxicity. Accordingly, no grade 3 or 4 late effects were observed so far. One patient developed a rib fracture 330 days after reirradiation within an overlapping region receiving a total dose of 95 Gy (1.8 Gy/fraction). Pain was mild to moderate and pain medication was only temporarily necessary. No cases with grade 3 or 4 plexopathy were observed so far.

## Discussion

We treated a heterogeneous group of 29 patients suffering from recurrent breast cancer after previous radiotherapy. Reirradiation was given with cumulative total doses of 80.4 to 126 Gy applied with or without concurrent chemotherapy. Palliation was good to excellent in all patients lasting for a long time of the patients' lifetime in the majority of cases. Bearing in mind the short median follow up, two infield recurrences were detected. Acute and late tolerances are good to excellent with no grade 3 or 4 normal tissue toxicity.

Techniques of reirradiation vary and may be applied with brachytherapy, intraoperative radiotherapy or external beam radiation. In general, literature on reirradiation in breast cancer is rare and not available on a systematic basis concerning radiation techniques.

Repeat high-dose partial breast irradiation after excision of an in-breast recurrence was offered 39 women who have refused mastectomy or were considered suitable for repeat lumpectomy with reirradiation by Deutsch [9]. The local recurrence-free survival was 76.9% after reirradiation with a median follow-up of 51.5 months, the overall survival 77.9% at 5 years. The distant metastases rate was 20.5%. The cosmetic results were excellent or good in 27 patients. In nine patients the cosmetic outcome was fair or poor.

Harms et al. [10] treated 58 patients with local recurrences (31 had concomitant distant metastases) after mastectomy and postoperative radiotherapy (mean dose 52 Gy). Retreatment consisted of pulsed-dose-rate brachytherapy (PDR brachytherapy) with two fractions of 22 and 18 Gy or twice 20 Gy separated by an interval of 30 days were applied. The two and 3 year locoregional recurrence-free survival rates for R2-resected tumours were 81% and 75%, for R1- resected tumours 85% and 71%, respectively. The majority of macroscopic tumour recurrences (R2) had a complete remission (28/30) after reirradiation. The normal tissue toxicity seemed to be rather high with grade 3/4 toxicity of 22% comprising in the majority of cases marked teleangiectasia and less often skin contracture. Pulsed-dose-rate brachytherapy was also used by Resch and colleagues [11] who re-treated 17 patients with small local recurrences with local excision and either a combination of PDR brachytherapy (total dose 12.5 – 28 Gy) and EBRT (total dose 12–30 Gy) or PDR brachytherapy alone. The initial radiation dose to the whole breast plus tumour bed was 50 to 60 Gy. The cumulative radiation was approximately 100 Gy as stated by the authors without details for individual patients. Four patients (24%) had second recurrences all within one year after retreatment and underwent mastectomy. No patient with PDR brachytherapy alone and retreatment doses of 40.2 to 50 Gy experienced a local recurrence. Biologically a dose of 40 to 50 Gy given within 4 days can be expected to be more efficient than a conventional fractionated EBRT given within five weeks. Skin and subcutaneous tissue side effects were moderate with no grade 3 or 4 toxicity. No unacceptable, i.e. deformities of the breast, results were observed and 5/17 had good to excellent results.

A case report of a 63-year-old female patient with left ductal breast cancer 13 years after primary treatment (mastectomy, axillary dissection, and 50 Gy postoperative irradiation) was published by Mayer at al. [12]. Perioperative HDR-AL with Ir-192 was performed with a dose per fraction of 6 Gy to the reference line, two fractions per week, to a total dose of 30 Gy. The tumour was locally controlled and the patient disease-free at five years. Skin toxicity did not exceed grade 2.

A novel technique to irradiate patients is intraoperative radiotherapy with a 50 kV X-rays source (Intrabeam™) treating patients during surgery and limiting radiation dose to the tumour bed. Kraus-Tiefenbacher et al. [13] reported on 17 patients with in-breast recurrences reirradiated with the Intrabeam™ source (median dose 20 Gy to the applicator surface). Note that the dose distribution by the Intrabeam™ source is characterized by a sharp dose fall off, i.e. in 1 cm depth from the applicator surface only 5 Gy are delivered. As the dose per fraction is high and the radiobiology is different from fractionated Megavoltage treatment with external beam irradiation, a direct comparison of both modalities is difficult. After a median follow-up of 26 months 16/17 patients were still alive, one patient died 26 months IORT due to pulmonary metastases (19 months after retreatment). Two other patients had distant metastases but were alive. No local recurrences were observed. Acute toxicity was mild with no Grade 3/4 toxicities and there was no delay in wound healing or wound infection. The cosmetic outcome was rated in the majority of cases as good to excellent, at least as fair.

The largest number of reirradiated patients were reported by Kapp et al. [14] and van der Zee et al. [15,16] in combination with hyperthermia. Kapp et al. reported on 89 patients of which about half had prior radiation therapy (the exact number was not given) with an average dose auf 50,6 Gy. These patients received reirradiation with a total dose of 35 to 40 Gy (dose/fraction 1.8 to 2.1 Gy) to the ecisional biopsy in conjunction with hyperthermia. The average cumulative total dose was probably 90 Gy though no details were reported on individual patients. Longer duration of local control was achieved with concurrent reirradiation doses greater 39,5 Gy. The local failure rate was 24% in the subset of patients with biopsies only.

J. van der Zee and colleagues first published in 1988 on ninety-seven patients with recurrent breast cancer previously irradiated [15]. Reirradiation without prior surgery was given twice weekly with 2 to 4 Gy per fraction to a total dose of 8 to 32 Gy. The high dose per fraction was probably given because of the palliative intention and the fact that most patients were heavily pre-treated with different kinds of chemotherapy and endocrine therapy. The cumulative dose was 40 to 96 Gy. Hyperthermia was applied concurrently. The time interval between first and second radiation therapy was 1 to 203 months, mean 48 months. A complete response was achieved in 35% and a partial response in 55%. In many patients no local progression occurred during their lifetime. The authors concluded that 8 × 4 Gy in conjunction with hyperthermia is safe and feasible without severe toxicity. In 1999 the authors published a second study [16] with longer follow up and more patients. The minimum time interval between first and second radiation course was 4 months. Again, a single dose of 4 Gy was given twice weekly concurrent with hyperthermia, total dose 12 to 32 Gy (129/134). A clinical complete response rate of macroscopic tumours of 71% was achieved. Within the group of completely responding tumours or reirradiation after R1-resection, approx. 30% recurred in-field after a median follow up of 11 months. The median duration of local control was 32 months. Moderate to marked erythema was observed in 48/134 patients, ulcerations in 14 patients (nine had tumour-induced ulcerations before start of retreatment).

Phromratanapongse and colleagues reported in 1993 on 44 patients with locally recurrent, previously irradiated adenocarcinoma of the breast [17]. Hyperthermia was combined with concurrent radiation doses varying from 16 to 56 Gy. In 41% of these inoperable recurrences a complete response could be achieved with low acute toxicity. Two had ulcerations with infection and one patient severe blisters.

Inoperable recurrent breast cancer in fifteen patients, who had undergone a radical mastectomy and conventional radiotherapy (60 Gy), were entered on a multimodal protocol consisting of initial treatment with radiotherapy and a monthly infusion of liposomal doxorubicin in conjunction with local hyperthermia treatment [18]. All patients received reirradiation up to a total dose of 30.6 Gy in 1.8 Gy/fraction. All patients showed an objective measurable response and 3 patients (20%) demonstrated a clinically complete response.

In the current study, part of the patients also received concurrent chemotherapy with either continuous infusion 5-FU or capecitabine (one case with docetaxel) in inoperable or incompletely resected patients. The rationale for choosing concurrent chemotherapy was the expectation of an additional locoregional. In head and neck cancer reirradiation combined with chemotherapy results in prolonged survival and long term survival in some patients [19]. As the current study was not a randomized one examining reirradiation alone with combined chemotherapy and reirradiation, we cannot quantify the additional effect of chemotherapy. We chose 5-FU or capecitabine because of extensive clinical experience in combined modality treatment and the low risk of excessive skin toxicity with these substances. Cisplatin or docetaxel, however, might well be good alternatives as these agents are widely used in the combination with radiotherapy e.g. in head and neck cancer.

## Conclusion

Including the results of this study, data on more than 300 patients reirradiated for recurrent breast cancer either alone or in combination with chemo-endocrine treatment and/or hyperthermia have so far been published. Reirradiation is feasible and save at least within the first five years after retreatment with low to modest side effects offering a second curative chance *with *breast conserving therapy. In palliative intended treatment, results are good to excellent lasting for most of the patients' lifetime. The minimum time interval between first and second radiation treatment should be six months based on published data although the exact time interval is unknown. The duration and percentage of local control is dose dependent. The minimum second radiation dose in fractionated irradiation should be 40 Gy though higher doses might be possible depending on the treatment volume. With regard to possible late side effects, we recommend a dose per fraction of 1.8 to 2 Gy in curative intent. Higher doses per fraction can be given in patients expecting a lifetime of only a few months. Reirradiation can be safely combined with continuous infusion 5-FU or oral capecitabine. Possible alternative radiation techniques to fractionated Megavoltage external beam therapy are brachytherapy and intraoperative radiation therapy.

## Competing interests

The authors declare that they have no competing interests.

## Authors' contributions

FW carried out the conception and design of the study, was responsible for the treatment of patients, performed the data acquisition, analyzed and interpreted the data. JD participated in the design of the study and treatment of patients, and helped to draft the manuscript. CP participated in the design of the study and treatment of patients, and helped to draft the manuscript. CW participated in the design of the study and was responsible for the treatment with chemotherapy. MK conceived of the study and was responsible for radiation treatment planning. CB conceived of the study and took part in radiation treatment planning. All authors read and approved the final manuscript.
